# The Impact of a Rights-Based Counselling Intervention to Reduce Stigma in People Affected by Leprosy in Indonesia

**DOI:** 10.1371/journal.pntd.0005088

**Published:** 2016-12-13

**Authors:** Mimi Lusli, Ruth Peters, Wim van Brakel, Marjolein Zweekhorst, Sorana Iancu, Joske Bunders, Irwanto, Barbara Regeer

**Affiliations:** 1 Centre for Disability Studies, Selo Sumarjan Research Centre (SSRC), Faculty of Social and Political Sciences, Universitas Indonesia, Depok, Indonesia; 2 Athena Institute, Faculty of Earth and Life Sciences, VU University Amsterdam, Amsterdam, The Netherlands; 3 Disability Studies, VUmc, Amsterdam, The Netherlands; 4 Netherlands Leprosy Relief, Technical Department, Amsterdam, The Netherlands; 5 EMGO+ Institute for Health and Care Research, VU Medical Centre and GGZ inGeest, Amsterdam, The Netherlands; Fondation Raoul Follereau, FRANCE

## Abstract

**Background:**

This paper assesses the impact of a counselling intervention on reducing leprosy-related stigma in Cirebon District, Indonesia. The unique features of this intervention are its rights-based approach, the underlying Cognitive Behavioural Therapy (CBT) model, the three types of counselling and the lay and peer counsellors who were involved.

**Methodology/principal findings:**

Mixed methods (e.g. three scales, interviews, focus group discussions and reflection notes) were used to assess the impact of the intervention, which ran over a two-year period. There was a control area with no interventions. The study participants were people affected by leprosy and other key persons (e.g. family members). The sample size differs per method, for example, data regarding 67 counselling clients and 57 controls from a cohort, and notes from 207 counselling clients were examined. The notes showed that most clients faced stigma on a daily basis, whether internalized, anticipated and/or enacted. A significant reduction was found between the before and after total scores of the SARI Stigma Scale (p-value < 0.001), Participation Scale Short (p-value < 0.001) and WHO Quality of Life score (p-value < 0.001) among the counselling clients. While there is also an effect in the control group, it is much larger in the intervention group. Qualitative data indicates that knowledge and rights trigger change. Clients took steps to improve their life such as re-connecting with neighbours, helping in household activities and applying for jobs. Challenges include the wish to conceal their condition.

**Conclusion/significance:**

The findings show that the counselling intervention was effective in reducing stigma, promoting the rights of people with leprosy and facilitating their social participation. More research is needed on how to create a more sustainable intervention, preferably structurally embedded in the health or social services.

## Author Summary

Can building knowledge, increasing the awareness of rights and developing confidence through counselling empower people affected by leprosy and reduce the stigma that surrounds them? In this paper the authors describe the impact of a counselling module designed to address the widespread issue of stigma in Cirebon District, Indonesia. The module ran for a two-year period and has some unique features. For example, it integrates three different types of counselling: individual, family and group. During the counselling, medical information about leprosy was provided and awareness of rights (e.g. right to healthcare, right to education) was raised. The counselling was offered by lay and peer counsellors, the latter having been affected by leprosy, to a total 260 people. This study showed that counselling can be effective in reducing stigma. The authors recommend its application on a larger scale.

## Introduction

*“My parents have always hidden my disease from me. I was eager to know, but nobody wanted to tell me. Even my health worker [did not tell me]. I was angry at myself. People around me just asked me to take rest and stay in my bedroom.”* (Girl 15 years old, Cirebon District, Indonesia)

The girl quoted above wanted to know what disease she had. Her parents and the health professional, however, decided that it was better not to tell her. The girl was affected by leprosy, which remains a stigmatized condition worldwide. Persons or their family who are affected by a stigmatized condition often try to conceal it [1–3]. There are, however, costs to concealment: the burden is carried alone, managing concealment is onerous and not being informed might evoke negative feelings and emotions in the persons involved (as happened with this girl) [4]. Disclosure, on the other hand, can also have negative consequences. Family or community members who know that someone around them is affected by leprosy might want to care, show kindness and support the person, but more often people are worried about infection and tend to distance themselves. Many studies in countries ranging from Bangladesh to Brazil, India, Indonesia, Nepal, Nigeria and Paraguay have shown that leprosy and leprosy-related stigma specifically can lead to negative feelings and emotions, restricted participation, discrimination, and thus a reduced quality of life [1,5–11]. Not surprisingly, the stigma attached to the disease is often a greater concern for those affected than the disease itself [12,13].

There is an increased awareness among researchers, policy makers and practitioners of the importance of addressing leprosy-related stigma. Reducing stigma will improve the lives of the people affected and will also assist in disease management and control. Of the many stigma-reduction interventions that have been identified, counselling has been described as a promising approach [14,15]. Counselling is defined by Yeo [16]–a counsellor from the tradition of Cognitive Behavioural Therapy (CBT)–as a collaborative process in which the counsellor or psychologist facilitates the expansion of people's view of life; enlarges their repertoire of coping resources; and enables them to make choices for change in themselves, the situation, and the environment without destructive consequences to the self or to others. Though promising, it is not easy to reduce leprosy-related stigma through counselling. Stigma is a complex problem. Its multiple causes are often deeply rooted in societal norms and values [12,17,18]. Moreover, the dynamics and interconnections between the causes and consequences of stigma can erode the effect of any intervention [19]. Studies have shown that interventions need to be context-specific, multi-targeted and oriented at different levels, but it remains unclear how this can be achieved by single interventions [14]. For a counselling intervention, specifically, the added challenge is often the lack of professional counsellors.

Recently, a new counselling practice named the Rights-Based Counselling Module (RBCM) has been developed in Cirebon District, Indonesia [58]. The approach imports human rights principles into the counselling sessions, especially the right to healthcare, to help empower the clients. Based on an exploratory study that aimed to understand the characteristics of people affected by leprosy and the views of the community, a draft module was developed, and piloted with 62 clients. The module applies CBT principles, integrates three types of counselling (individual, family and group) and is knowledge- and rights-based. The idea is that five counselling sessions can trigger clients affected by leprosy to move from a seemingly hopeless situation into a space where they feel hope, take initiatives and experience less internalized stigma. A unique feature of this intervention is that stigmatized individuals are trained and involved as lay and peer counsellors.

Lusli et al. [58] concluded that RBCM seems a promising approach to reducing leprosy-related stigma. It might be asked, however, whether offering three types of counselling istoo ambitious, whether lay and peer counsellors are appreciated as counsellors, whether an awareness of rights is as powerful as anticipated, whether an emphasis on knowledge—which is often critiqued in the field of stigma reduction [20]–will work, and what are the likely challenges. This study aims to assess the effect of this counselling intervention on stigma experienced by people affected by leprosy in Cirebon District, Indonesia.

### Theoretical framework

In the past, stigma was primarily considered an attribute; a legacy of Goffman’s seminal work [21], or of its interpretation. The current emphasis lies much more on stigma as a social process and thus goes beyond the individual body [22]. A well-known definition of health-related stigma that we employ in this study is:

*… a social process*, *experienced or anticipated*, *characterized by exclusion*, *rejection*, *blame*, *or devaluation that results from experience*, *perception or reasonable anticipation of an adverse social judgment about a person or group*. [23]

To distinguish different types of stigma, Weiss [24] extended the Hidden Distress Model of Scambler [25], and identified three types for those who stigmatize and three for those who are stigmatized. This paper focuses mainly on the types of stigma faced by those who are stigmatized: anticipated, internalized and/or enacted [25,26]. The latter refers to the experience of discrimination, and is also called experienced stigma. Anticipated or perceived stigma is the fear of being discriminated against. Finally, internalized or self-stigma is the stigma people apply to themselves due to negative views about the self, which could lead to feelings of shame and guilt. It is an internalized perception of being devalued or "not as good as" another individual, and is seen as a source of anguish and unhappiness [25]. A different categorization of stigma is based on three levels: structural, social and individual [27–30], and this paper considers the social level (interpersonal) and individual level (intrapersonal).

It is not easy to assess a concept as complex as stigma [20]. We decided to assess experiences of stigma, and two aspects that are negatively affected by it: *participation* and *quality of life*. Studies in India, the Netherlands and the Philippines have shown that leprosy and, in particular, leprosy-related impairments, can negatively affect one’s social participation [31–33]. This association was studied and confirmed in Indonesia. Van Brakel et al. [7] found that 60% of the people with a leprosy-related impairment experience restrictions on their participation. Quality of life is another well-known overall measurement. The effect of leprosy or leprosy-related stigma on the quality of life is interesting but has been little studied, and when it has, the results are mixed. Brouwers et al. [34] did not find a significant association in a multivariate analysis on data from East Nepal, while Tsutsumi et al. [5] did find an association between anticipated stigma and quality of life in Bangladesh. By assessing stigma, participation restriction and quality of life we hoped to gain a broad impression of the impact of the counselling intervention on stigma.

## Methods

### Study design

This study is part of the Stigma Assessment and Reduction of Impact (SARI) project conducted in Cirebon District, Indonesia (2010–2015). The SARI project aimed to assess the effectiveness of three stigma-reduction interventions in persons affected by leprosy in Cirebon District, West-Java, Indonesia: counselling, contact [35] and Social Economic Development (SED). The SARI project is a cluster-randomized controlled intervention study and uses the Interactive Learning and Action approach [36] as a guiding methodology.

### Research team

The SARI team is interdisciplinary and inclusive—the scientific staff comes from a range of disciplines and several team members are affected by leprosy or have a disability. For instance, the first author (ML) has a visual disability, the principal investigator (I) uses a wheelchair, and four of the ten local research assistants have a disability or have been affected by leprosy. The SARI team works closely with local, provincial and national Health Offices and with a local Disabled People’s Organisation (DPO).

### Study area

Cirebon District was selected as the study area because it has a relatively high number of new cases annually and—according to national experts—a higher level of leprosy-related stigma than in other districts and no interventions to address this. Thirty sub-districts of Cirebon District were randomly allocated a paired intervention or became a control area where no interventions were made. The interventions areas included: (i) ‘Counselling—Contact’; (ii) ‘Contact—SED’; (iii) ‘SED—Counselling’; and (iv) ‘Control’. The baseline study was conducted in 2011, the counselling intervention ran from January 2012 to December 2013 and the final survey was made in the second quarter of 2014. This enables us to assess a relatively long-term impact.

### Study population

The study population included people affected by leprosy living in the area where the interventions were offered. Data was also collected regarding current counselling clients, their family members, health professionals, lay and peer counsellors and SARI’s research assistants to get a rich perspective on the effect of the intervention and to enhance the validity of this study.

### The counselling intervention

The counselling intervention was developed during the first year of the project and the idea was that it would address stigma primarily at the individual, and secondarily at the social level. An exploratory study was conducted (see [3,10,37]) and based on its findings a counselling practice was drafted and piloted, which led to the Rights-Based Counselling Module (RBCM) (see Box 1). This module can be managed by lay and peer counsellors. The SARI project selected 28 people as potential lay and peer counsellors, including the project’s ten research assistants. They attended 56 hours of RBCM training, and eventually 23 became a counsellor (15 men and eight women; ten were affected by leprosy, six have a physical disability, one has a visual impairment and six had no disability or leprosy). They worked in teams of three and were supervised by the first author (ML) (for more details on the selection, training and perceptions of lay counsellors [38]).

**Box 1: Rights-Based Counselling Module [58]**

**Counselling is given by**

Lay and peer counsellors

**Key principles**

1. Every client, whatever their condition, wants to change their life for the better and should decide what actions/ solutions are needed to bring about this change.
2. Each client needs to be listened to, appreciated and acknowledged.
3. In a relaxed, though energetic, fun and joyful atmosphere, the client will be comfortable and more open and trust will come more easily.
4. Medical knowledge about leprosy is a prerequisite for the rest of the counselling process.
5. Awareness of rights is the basis for developing confidence, making changes in life and participating in society.

**The 5C framework**

The 5C framework describes five important counselling skills (confirmation, clarification, confrontation, compromise and commitment) and puts these skills in a certain order.

**Content of the five sessions**

Session 1: Assessment of situation and trust building, individual counselling, 30–45 minutes
Session 2: Knowledge, rights and dealing with stigma, individual counselling, 30–45 minutes
Session 3: Knowledge and solutions in the family context, family counselling, 30–45 minutes
Session 4: Learning from each other and action, group counselling (4–6 clients), 45–60 minutes
Session 5: Sharing and strengthening action, group counselling (4–6 clients), 45–60 minutes

In total, 260 persons affected by leprosy were offered counselling: 62 during the pilot phase and 198 during RBCM phase. The counselling offered during the two phases was broadly similar in terms of counselling types and style, but adjustments in, for example, the number of sessions, were made to make it more appropriate and therefore more effective. Of these 260 persons, 53 (20.4%) decided during the first session that they did not need or want to receive counselling. Reasons given by these 53 persons included no or limited stigma and fear for disclosure. The remaining persons became the counselling clients (n = 207). The number of sessions and type of counselling differed by client and depended on their needs and wishes (see Table 1).

**Table 1:** Overview counselling sessions.

Phase	Counsellor	Module	Session	Counselling type
Pilot	ML	Draft counselling module	1	Individual
			2	Individual
			3	Individual
			4	Individual
			5	Family
			6	Family
			7	Group
			8	Group
RBCM phase	Lay and peer counsellors	RBCM	1	Individual
			2	Individual
			3	Family
			4	Group
			5	Group
			> 5	Individual

### Research methods

Mixed methods were used to get an in-depth understanding of the effects of the counselling and how these were achieved (see questions in introduction) and to still be able to generalize the findings. Three scales were used: the SARI Stigma Scale (SSS), Participation Scale Short (PSS) and the World Health Organization Quality of Life instrument (WHO-QOL BREF). The SSS aims to assess stigma and is based on the HIV Stigma Scale developed by Berger et al. [39]. The scale has 21 items (score 0–3, min-max total score 0–63) and four domains: experienced stigma (min-max total score 0–21), disclosure concerns (min-max total score 0–12), internalized stigma (min-max total score 0–18) and anticipated stigma (min-max total score 0–12). The cross-cultural validity of the SSS was tested in Cirebon District and found to be adequate for the Bahasa Indonesia-speaking population [59]. The Participation scale assesses participation restrictions and is based on the Participation domain of the International Classification of Functioning, Disability and Health [40]. The validity of this scale has been tested and found to be adequate in several Asian countries [40–42]. A shortened version of the Participation scale, the Participation Scale Short, has 13 items (score 0–5, min-max total score 0–65) [43]. This is the version we applied. The WHO-QOL BREF instrument is a shorter version of the original WHO-QOL instrument, comprising 26 items (score 1–5, min-max total score 26–130), which measure the broad domains of physical health, psychological health, social relationships and environment. The validity has been tested and was found to be adequate in an Indonesian-speaking sample [44]. Some of the domains and items of these scales are more relevant than others. Of particular interest are, for instance, the SSS internalized stigma and the psychological health domain of the WHOQOL-BREF.

Applying multiple instruments can be quite burdensome for respondents. If the interviewer noted that a respondent was tired and not so keen on continuing the interview, they were instructed to drop the WHO-QOL BREF. Based on a sample-size calculation and an anticipated loss to follow-up it was decided that 600 people affected by leprosy had to be part of the quantitative part of the baseline. Health professionals invited people affected by leprosy (currently in treatment or cured) to different health clinics for an interview.

In addition, in-depth interviews (IDI) and Focus Group Discussions (FGD) were applied using purposive sampling to ensure adequate representation of men and women, different age groups and intervention areas. For the IDI in the final survey, clients whose lay or peer counsellors expected positive outcomes as well as those with limited or no positive outcomes were selected. We aimed for 80 interviews for the baseline and 25 for the final survey. As many paired interviews as possible were conducted (same interviewee for baseline and final survey). The IDIs aimed to gain insight in the extent of stigma in people affected by leprosy before and after the intervention. The topics addressed were: leprosy history, feelings, family and friends, community, economic condition, and future. The FGDs aimed to assess the impact of the counselling after the intervention. Different groups of participants joined these discussions including counselling clients and their family members, health professionals, lay and peer counsellors and research assistants. Topics of the FGD were changes that occurred due to counselling, influence of the type of counselling, role of the type of counsellor and strengths and weaknesses of the counselling.

Finally, two types of notes were prepared. The Participant Reflection Notes (PRN) were written at the end of the counselling and aimed to identify the benefits of counselling for the clients. Clients received nine questions to guide their reflections (e.g. changes experienced, remaining expectations from counselling). These notes were not written during the pilot but only during the implementation of the RBCM. The Counsellor Reflection Notes (CRN) aimed to provide insight into the types of stigma the client experiences, if and what changes occurred during the counselling sessions and how counselling might have contributed to these changes. Table 2 provides an overview of all the methods.

**Table 2:** Overview research methods applied in this study.

Research methods	Baseline (2011)	Implementation counselling (2012–2013)	Final survey (2014)
Participation Scale Short (PSS)	x		x
SARI Stigma Scale (SSS)	x		x
WHO-Quality of Life (WHO-QOL BREF)	x		x
In-depth interviews (IDI)	x		x
Focus Group Discussions (FGDs)			x
Participant reflection notes (PRN)		x	
Counsellor reflection notes (CRN)		x	

### Data management and data analyses

The interviews and FGDs were recorded, transcribed and translated into English. This data was analysed by ML. The quantitative data was entered into an Epi Info for Windows database (version 3.5.3) and analysed using Stata 12.1 by RP. Demographic variables included sex, age (in years), married (yes/no), education, disability grade (0/1/2) (using the WHO leprosy disability grading system [45]. This paper addresses the impact of the counselling intervention as a whole and not the impact of its individual activities. Therefore, all participants who were part of either the pilot counselling given by ML or of the RBCM given by lay and peer counsellors were combined in one group for the main analysis. To investigate the effect of the interventions, we calculated means, SD, and performed simple regressions (t-test, paired t-test, Wilcoxon matched-pairs signed-ranks test). P-values less than .05 were taken as significant.

### Ethical considerations

Permission to undertake the study was obtained from the relevant government offices. Written informed consent was obtained from individual study participants. The control area in this study was a “care-as-usual” area.

## Results

### Socio-demographics study participants

**Fig 1:**
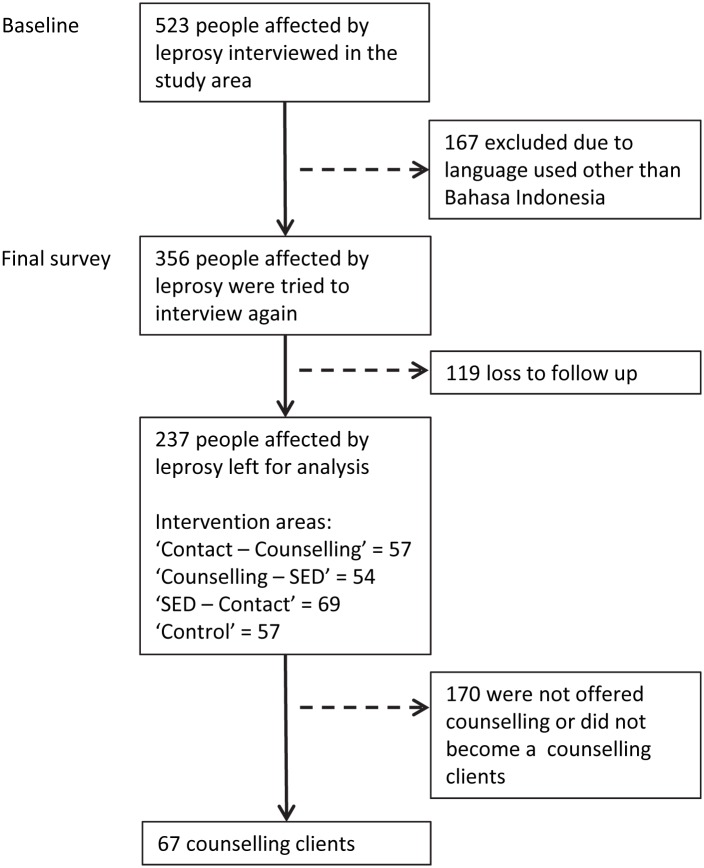
Selection procedure.

Scales were administered in 523 people affected by leprosy living in the study area during the baseline. For the final survey only people affected by leprosy whose interview was administered in Bahasa Indonesia and whom we were able to interview again were included. This resulted in 237 matched observations (see Fig 1). Given the number lost to follow-up we compared the 237 observations with those who could not be interviewed again to see if there were any systematic differences that might indicate bias (Table 3). No significant differences were found. As shown in Fig 1, of these, 111 (57+54) people affected by leprosy lived in the areas where counselling was offered. In total, 67 of the cohort joined the intervention: 23 received counselling during the pilot from ML (of which seven were selected and also became peer counsellors) and 44 received counselling from lay and peer counsellors using the RBCM. Of the 67 clients, 18 also participated in SED-related activities (received microcredit, attended a training or received livestock) and 34 lived in areas where events were organised that aimed improve negative community perspectives and attitudes. The socio-demographic characteristics of the participants of the counselling intervention are shown in Table 4. Also more detailed information about the differences between male and female (the men affected by leprosy in this cohort are less often married and have a higher level of education) are provided.

**Table 3:** Socio-demographic characteristics people affected in cohort.

Variables		Cohort (n = 237)	Observations not part of cohort (n = 119)	P-value*
Sex	Female; n (%)	97 (40.9%)	37 (31.9%)	0.098
Age (in years); mean (SD)		36.5 (14.0)	34.2 (13.5)	0.143
Marital status	Married; n (%)	162 (68.4%)	77 (65.3%)	0.557
Education	No education; n (%)	17 (7.2%)	6 (5.0%)	0.190
	Primary school; n (%)	143 (60.3%)	63 (52.9%)	
	Secondary school; n (%)	77 (32.5%)	50 (42.0%)	

*Overall group differences, based on t-test for continues variables and X2 statistics for categorical variables.

**Table 4:** Socio-demographic characteristics counselling clients from the cohort.

Variables		Cohort (n = 67)	Male (n = 36)	Female (n = 31)
Sex	Female; n (%)	31 (46.3%)	--	--
Age (in years); mean (SD)		34.9 (13.8)	34.6 (15.5)	35.2 (11.8)
Marital status	Married; n (%)	45 (67.2%)	18 (50%)	27 (87.1%)
Education	No education; n (%)	3 (4.5%)	1 (2.8%)	2 (6.5%)
	Primary school; n (%)	40 (59.7%)	18 (50%)	22 (71.0%)
	Secondary school; n (%)	24 (35.8%)	17 (47.2%)	7 (22.6%)
Disability grade	0	26 (38.8%)	13 (36.1%)	13 (42.0%)
	1	33 (49.3%)	18 (50%)	15 (48.4%)
	2	8 (11.9%)	5 (13.9%)	3 (9.7%)

Seventy-seven IDI were conducted during the baseline and 24 during the final survey; five were paired. Of these 77 IDI, 38 were with women and 39 with men; mean age was 32 (youngest was 16 and eldest was 70). Of the 24 IDI in the final survey, 14 were with women and 10 with men; the mean age was 44 (youngest was 18 and oldest was 70). There were nine FGD in the final survey with a total of 64 participants (see Table 5 for a detailed overview). PRN were written by 145 clients and CRN were written by ML and 12 lay and peer counsellors on the counselling sessions of 207 clients (five went missing).

**Table 5:** Overview of FGD.

FGD ID	Participants	Group size	Males	Females
1	Clients women	3	-	3
2	Young clients	5	3	2
3	Elderly clients	6	5	1
4	Clients with impairments	9	8	1
5	Clients who also joined the SED intervention	7	2	5
6	Family members of clients	8	2	6
7	Lay and peer counsellors	10	5	5
8	Health professionals	8	7	1
9	Research assistants	8	7	1

### Different types of stigma at the start of counselling: internalized, anticipated and enacted

The CRN notes show that most clients faced daily internalized, anticipated and/or enacted stigma. Usually they faced combined types of stigma, but often one type was dominant. According to the CRN of the 202 clients 93 (46%) dealt mostly with internalized stigma, 54 (27%) mostly with anticipated stigma, 38 (19%) mostly with enacted stigma, while 17 (8%) experienced no stigma.

Those facing *internalized stigma* mentioned that they felt shame, were worried, felt dirty because of the lesions on their face and body, feared impairment, and had lost confidence. As a result, some of them opted to conceal their disease from their family, decided to stop working, preferred to stay at home, did not want to meet people and rejected invitations. About 5% of them admitted to having had suicidal thoughts. Most of those who experienced *anticipated stigma* feared being excluded and/or suffering discrimination. These clients wondered whether leprosy can be transmitted and whether it can be cured. Again a wish to conceal was found in this group. Those who experienced *enacted stigma* said they were treated badly by family and community members, which restricted their participation in their daily lives. Some had to stop going to school, lost their job, and lost their family and friends. Table 6 provides sections from the CRN notes to illustrate how each type of stigma manifested itself. Occasionally the counsellors concluded that the client experienced little or no stigma. While these clients also faced negative attitudes they dealt with these in a very positive way, they were full of spirit, accepted their disease, did not care about what others said about them and had sufficient medical information about leprosy.

**Table 6:** Pieces from CRN connected to types of stigma.

Type of stigma	CRN notes:	Client
Internalised	“He is ashamed of his disease so he hides himself and sleeps all day in his bedroom” (CRN12.103)	Man, 17 years
Internalised	“She is very ashamed of her disease and she wants to commit suicide” (CRN2.9)	Woman, 47 years
Internalised	“He is feeling useless, ashamed of himself (…) he is hiding from people” (CRN7.82)	Man, 30 years
Anticipated	“He feels despair because he believes that his disease is not curable, he is worried that he cannot support his children” (CRN2.8)	Man, 58 years
Anticipated	“He was afraid his health condition would get worse (…) he was afraid people would mock him” (CRN8.90)	Man, 30 years
Anticipated	“She feared being avoided by her husband, she lied when she took medicine, she said it was for an allergy” (CRN5.56)	Woman, 30 years
Enacted	“Due to leprosy he stopped going to school, his friends rejected him (…) they did not want to sit near him” (CRN2.10)	Man, 18 years
Enacted	“He was excluded by people in the farm, they made him stop working there, he was frustrated” (CRN1.3)	Man, 58 years
Enacted	[Because of leprosy] “she was asked by her mother-in-law to get a divorce” (CRN12.138)	Woman, 39 years
No/ limited stigma	“He is fine, his friends know about his disease, he is not afraid since he still has his friends for hanging out together” (CRN4.42)	Man, 25 years
No/ limited stigma	“She is okay meeting and talking to her neighbour, she does her daily work without worry, she has been cured for a long time” (CRN7.84)	Woman, 34 years

This categorization is a simplification of the clients’ complex reality. As mentioned, most clients dealt with a combination of types of stigma at the same time:

*“I am ashamed of my body, keeping silence is better. If I go for work, people will exclude me and stop me from doing the work because I am dirty and my disease is a danger for them. I am afraid that people will reject me.”* (IDI8, Man, 52 years)

*I feel disappointed, I have leprosy. I blame myself, it will kill my future. When I go out, my neighbours always discriminate and avoid me, I am sad I do not have neighbours, I am afraid to feel alone.”* (IDI21, Woman, 55 years)

This analysis of the qualitative baseline data shows that stigma is a real, important and complex problem for many, but not all, people affected by leprosy.

### Impact of the counselling intervention

#### Total scores of SSS, PSS and WHOQOL-BREF

The analysis of the SSS, PSS and WHOQOL-BREF data shows that there are large and significant differences between the total scores at baseline and at the final survey in the counselling clients. The SSS total reduced from 21.55 to 12.00 (p-value <0.001), the PSS from 9.51 to 5.86 (p-value <0.001), and the WHOQOL-BREF increased from 80.19 to 86.74 (p-value <0.001) (see Table 7).

**Table 7:** Univariate difference between baseline and final survey and between counselling clients and controls.

		Baseline (2011)Mean (SD)	Final survey (2014) Mean (SD)	P-value	Difference Mean (SD)	P-value^3^
SSS total score	Counselling clients (n = 67)	21.55 (13.51)	12.00 (11.02)	<0.001*^1^	-9.55 (12.69)	0.086
	Control (n = 57)	15.42 (11.11)	9.79 (10.97)	<0.001*^1^	-5.63 (12.39)	
PSS total score	Counselling clients (n = 67)	9.51 (1.43)	5.86 (1.27)	<0.001*^1^	-3.65 (1.02)	0.091
	Control (n = 57)	5.42 (0.82)	4.05 (0.95)	0.052^1^	-1.36 (0.83)	
WHOQOL-BREF total score	Counselling clients (n = 67)	80.19 (1.14)	86.74 (1.4)	<0.001*^2^	6.54 (1.65)	<0.001*
	Control (n = 57)	85.83 (1.25)	83.83 (1.28)	0.264^2^	-2.00 (1.77)	

^1^. Wilcoxon matched-pairs signed-ranks test

^2^. Paired t-test

^3^. Two sample t-test

Comparing the counselling clients with the control group revealed large differences at baseline. The counselling clients experience more stigma (SSS total score of 21.55 versus 15.42), more participation restrictions (PSS total score of 9.51 versus 5.42), and have a poorer quality of life (WHOQOL-BREF total score of 80.19 versus 85.83) compared with the controls. In the controls a significant reduction between the baseline and final survey SSS total scores (p-value <0.001) and a nearly significant reduction in PSS total scores (p-value 0.052) were found. The differences in the counselling clients are, however, much larger than those found in the controls. The mean different SSS score was -9.55 in the counselling clients and -5.63 in the controls, which is not significant (p-value 0.086). Likewise, the mean different PSS score was -3.65 in the counselling clients and -1.36 in the controls (p-value 0.091).

The WHOQOL-BREF scores show a different picture. First, in the control group we found a reduction in WHOQOL-BREF total score between baseline and final survey (p-value 0.264), and an improved quality of life in the counselling clients. The mean difference in the controls was -2.00 and in the counselling clients, 6.54. This difference was significant (p-value <0.001).

We tested if there was an effect of several key variables (sex, age, education, marital status and disability grade) on the total scores. We found a significant effect of the variable of sex on the SSS and the PSS and decided to present the results separately (see Table 8). First, at baseline the women were worse off, with a higher SSS total score (greater stigma) and a slightly higher PSS score (more participation restrictions). Second, the counselling intervention reduced stigma and participation restrictions more in women than in men: -13.35 for women and -6.28 for men in the SSS total score (p-value 0.022) and -6.00 for women and -1.69 for men for the PSS total score (p-value 0.034).

**Table 8:** Results SSS and PSS presented for men and women counselling clients (n = 67).

		Baseline (2011) Mean (SD)	Final survey (2014) Mean (SD)	P-value^1^	Difference Mean (SD)	P-value^2^
SSS total score	Women (n = 31)	23.16 (2.53)	9.81 (1.72)	<0.000	-13.35 (2.05)	0.022*
	Men (n = 36)	20.17 (2.1)	13.89 (1.99)	0.009	-6.28 (2.16)	
PSS total score	Women (n = 31)	9.63 (1.58)	3.63 (1.11)	<0.000	-6.00 (1.26)	0.034*
	Men (n = 36)	9.42 (2.28)	7.72 (2.09)	0.020	-1.69 (1.47)	

^1^. Wilcoxon matched-pairs signed-ranks test

^2^. Two sample t-test

We also analysed the data separately for the pilot clients and the RBCM clients (see Table 9). The n per sub-group is too small to draw firm conclusions but we can see some trends. The differences in the SSS, PSS and WHOQOL-BREF are broadly comparable between pilot clients and RBCM clients; the difference is slightly larger in the pilot clients. The group with a different outcome is the pilot clients who eventually became peer counsellors. They have a larger reduction of the SSS total score, but a smaller reduction in the PSS and WHOQOL-BREF.

**Table 9:** Detailed results SSS, PSS and WHOQOL-BREF for counselling clients by sub-group (n = 67).

		Baseline (2011) Mean (SD)	Final survey (2014) Mean (SD)	Difference Mean (SD)
SSS total score	Counselling clients (n = 67)	21.55 (13.51)	12.00 (11.02)	-9.55 (12.69)
	Pilot clients + Peers(n = 23)	19.60 (2.83)	9.04 (1.57)	-10.56 (2.56)
	Pilot clients only (n = 16)	18.69 (3.62)	9.13 (1.60)	-9.56 (3.32)
	Peers counsellors only (n = 7)	21.71 (4.49)	8.86 (3.88)	-12.86 (3.81)
	RBCM (n = 44)	22.56 (2.04)	13.54 (1.85)	-9.02 (1.96)
PSS total score	Counselling clients (n = 67)	9.51 (1.43)	5.86 (1.27)	-3.65 (1.02)
	Pilot clients + Peers (= 23)	8.95 (1.66)	3.86 (1.10)	-5.09 (1.3)
	Pilot clients only (= 16)	9.56 (2.03)	3.88 (0.75)	-5.69 (1.76)
	Peer counsellors only (n = 7)	7.57 (3.01)	3.86 (3.37)	-3.71 (1.96)
	RBCM (n = 44)	9.81 (2.01)	6.93 (1.85)	-2.88 (1.37)
WHOQOL-BREF	Counselling clients (n = 67)	80.19 (1.14)	86.74 (1.4)	6.54 (1.65)
	Pilot clients + Peers (= 23)	80.95 (1.70)	88.55 (2.77)	7.6 (3.03)
	Pilot clients only (= 16)	81.14 (2.00)	90.71 (3.70)	9.57 (3.85)
	Peer counsellors only (n = 7)	80.50 (3.48)	83.5 (2.63)	3.00 (4.52)
	RBCM (n = 44)	79.78 (1.51)	85.75 (1.69)	5.97 (1.98)

The large and significant differences found between baseline and final survey in the counselling clients will now be explored in more detail by analysing the domains and items from the scales and by making use of the qualitative data.

#### Understanding the impact on stigma in more detail

Before exploring the differences we should underline that the counselling had limited or no impact on some clients. The qualitative data shows that these clients had either long been cured or were less affected by stigma than others. From the FGDs and IDIs it became clear that they nevertheless did enjoy the company of the counsellors.

*“I have been cured for a long time, but I like that you come and visit me. During your visit, I have a friend to chat with, and through some visits I just get information related to my health. It is good to remind me.”* (IDI11, Woman 55 years)

*“The visit that you call counselling*, *I like it*. *I enjoy your company rather than being alone at home*. *Please come whenever you want.”* (FGD3 Elderly clients)

To understand stigma at the individual level we used the four domains of the SSS. Significant or nearly significant differences were found between baseline and final survey in the experienced stigma domain (p-value < 0.011), disclosure concerns domain (p-value < 0.001), internalized stigma domain (p-value < 0.001) and the anticipated stigma domain (p-value 0.054) (see Table 10).

**Table 10:** Mean SSS domains scores for counselling clients (n = 67).

	Baseline (2011) Mean (SD)	Final survey (2014) Mean (SD)	P-value^1^	Difference Mean (SD)
Experienced stigma (min 0, max 21)	3.25 (0.62)	1.76 (0.49)	0.011*	-1.49 (0.55)
Disclosure concerns (min 0, max 12)	6.00 (0.48)	3.33 (0.39)	< 0.001*	-2.67 (0.55)
Internalized stigma (min 0, max 18)	6.79 (0.51)	3.36 (0.40)	< 0.001*	-3.43 (0.51)
Anticipated stigma (min 0, max 12)	4.25 (0.53)	3.04 (0.36)	0.054	-1.21 (0.55)

^1^. Wilcoxon matched-pairs signed-ranks test

**Fig 2:**
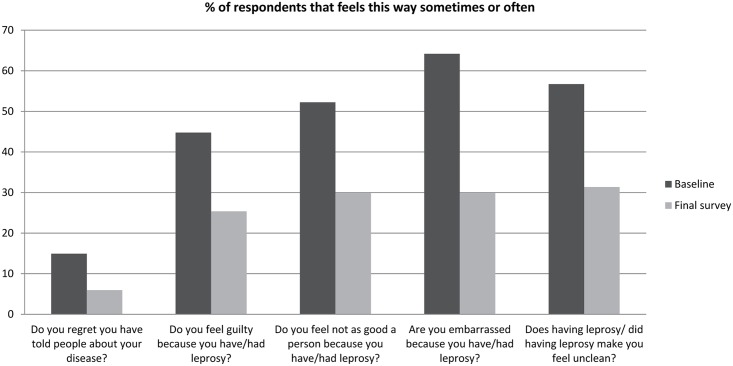
Changes in the Internalized stigma domain of the SSS before and after counselling (n = 67).

We studied in detail the changes that occurred in the Internalized stigma domain. Reductions were seen in all areas, but mainly in the items that ask about “embarrassment” (from 46.2% who often or sometimes felt embarrassed to 29.9%), in “feeling unclean” (from 57.7% who often or sometimes felt unclean to 31.4%) and in “feeling not as good a person as others” (from 52.2% who often or sometimes felt this to 29.9%) as shown in Fig 2.

**Fig 3:**
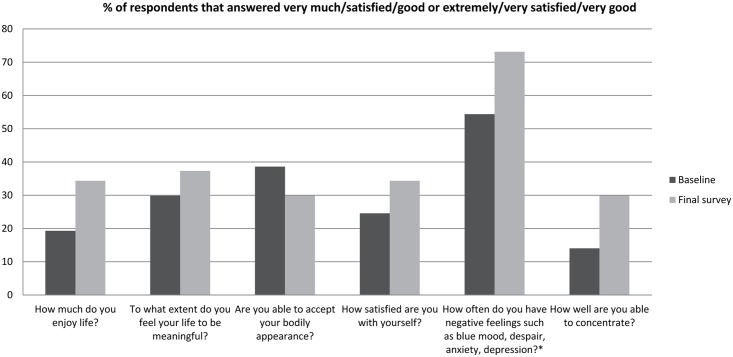
Changes in psychological health domain of the WHOQOL-BREF before and after counselling (n = 67) * % of respondents that answered never or seldom.

The psychological health domain of the WHOQOL-BREF is relevant in this context, showing a significant difference (p-value 0.014) between the baseline and final survey (see Table 11). The biggest improvement was in the item “negative feelings” (going from 54.4% who never or seldom felt negative feelings to 73.1%), but there were also improvements in “ability to concentrate” and “enjoy life” (see Fig 3). The findings on “accepting bodily appearance” were reduced, but the positive effect is partly hidden because two categories were merged. Before the intervention 33.3% indicated they were “mostly able” to accept their physical appearance and this was reduced to 7.5%, but 5.3% were “completely able” to accept their physical appearance which rose to 22.4%.

**Table 11:** Mean WHOQOL-BREF domains scores for counselling clients (n = 67).

	Baseline (2011) Mean (SD)	Final survey (2014) Mean (SD)	P-value^1^	Difference Mean (SD)
Physical health (min 0, max 21)	22.23 (0.41)	24.16 (0.48)	0.001*	1.93 (0.55)
Psychological health (min 0, max 21)	19.26 (0.35)	20.77 (0.38)	0.014*	1.51 (0.45)
Social relationships (min 0, max 21)	9.63 (0.21)	10.02 (0.21)	0.199	0.39 (0.30)
Environment (min 0, max 21)	23.25 (0.40)	25.00 (0.49)	0.005*	1.75 (0.60)

^1^. Paired t-test

The paired IDI at the baseline and during the final survey provided in-depth insights into the impact of the counselling intervention on stigma. The differences between feelings, emotions and experiences before the counselling (e.g. sadness, embarrassment, shame, fear and isolation) and after (e.g. confidence, happiness, knowledge, comfort, understanding) are illustrated in the Table 12.

**Table 12:** Before and after results of five clients paired IDIs.

Interviewee	Before receiving counselling (baseline 2011)	After receiving counselling (final survey 2014)
IDI10 Man 19 years	“I do not want to meet people as they gossip on me”	“I do not care what people are saying” “I am fully confident going out and meeting many people”
IDI2 Man 22 years	“I hide my disease as people always treat me as an ill person” “I am sad”	“I completely know my disease … I feel ok now to talk to people” “I am aware even though I still have medical treatment, … I have to do something for my future”
IDI7 Woman 44 years	“I feel embarrassed because of the spot in my skin” “I feel dirty”	“I feel happy … I met a woman who has the same disease as me” “I am back to do my daily works without confusion and wondering”
IDI12 Woman 45 years	“I always excuse using cosmetics to cover the spots on my face” “I am ashamed of it”	“I am ready and I am feeling comfortable going out without worrying about the spots”
IDI15 Woman 33 years	“I am afraid my disease will transmit to my family” “I isolate myself”	“I understand my disease well” “I am confident to explain my disease to other”

The PCN, CRN, IDIs and FGDs show similar changes. In general, the counselling clients were less shy and ashamed because of their disease and moved from a negative self-image towards a more positive one. The notes also show that clients experienced less worry or fear of meeting people or encountering people who stare or gossip. Finally, the notes show that clients were less affected and more capable of responding positively to experiencing discrimination and social exclusion. Clients realised they had the power to bring about these changes themselves. Also family members became aware of their role. Some quotes from the IDIs and FGDs illustrate this attitudinal change:

*“A client (…) always locked himself in the house because he was afraid of the insults from his neighbours, but after he received five full sessions of RBCM, he was able to change his mind. He positively dealt with his feelings, he was not afraid to meet and talk with his neighbours anymore. When I visited him he told me that he was appointed as an event organizer.”* (FGD9 research assistant)

*“After we received counselling we became more confident and independent*. *I am now positive about myself*, *since negative feelings and thoughts are bad for my future*.*”* (FGD4 clients with impairment)

*“Counselling really helped me and gave me personal motivation. Before receiving counselling, I locked myself inside the house and I stigmatised myself as a useless person. However, after receiving counselling, I am aware that my understanding of leprosy was wrong, I understood that leprosy cannot be cured, today I learned that leprosy can be cured.”* (IDI1, Man 46 years)

Changes also occurred at the social level. A stimulating and supportive family environment was created, mainly through family counselling. The CRNs show that during this counselling family members became involved. The medical information about leprosy was very important in helping family members be intimate again.

*“From the family counselling, we learned and we are completely sure now that leprosy is a disease that can be cured and is not contagious after having routinely taken medical treatment. The peer counsellor convinced us to change our perspective, to not fear to touch him and to include him in our family activities.”* (FGD6 family members)

The clients became more confident to take action and participate in their family. For instance, they started to ask questions (e.g. whether they could help with household chores, asked if somebody wanted to accompany them to the community health service) and family members in general responded positively to these changes:

*“My husband is now very active, out from his hiding place, full of spirit, helping me to make and sell crackers. I am really amazed that my husband wants to deal with many people without fear and worry.”* (FGD6 family members)

*“Due to counselling, I [now] prefer meeting people to do some work rather than just passively staying at home. I asked my father whether I can work with him on the farm taking care of the chickens. At noon, I visit my friends, joining with them to do community activities such as weekly praying, doing social events. I particular become more confidence trying to sell credit vouchers for mobile phones.”* (IDI17, Man 23 years)

Counsellors energetically encouraged the clients gradually to get involved in reducing stigma by participating in daily activities and to take initiatives. By getting out of the house, visiting the market, buying something at a shop, greeting neighbours, the clients realised that not everybody was looking at them (often nobody) or deliberately excluding them. Jobs, education, family relationships are important areas through which to increase participation, as illustrated in these quotes.

*“When I have a conversation, I agree that I should talk and I should reduce stigma. Leprosy has gone, stigma must stop, and I must get a better life. Counselling has motivated me to be brave to have an opinion and to take a decision to continue my study for my future career.”* (PCN57, Man 19 years)

*“Confidently I go out from home to participate in a social event, and people do not avoid me, they want to sit beside me, my perception was absolutely false.”* (PCN62, Man 47 years)

*“After I received counselling, at the last session, I changed my mind, I cannot sit for a long time. Seemingly there is high energy in my life that encourages me to do something. A few weeks after full counselling sessions, I re-opened my food shop in front of the house (…) The shop gives me money and gives me my life back. With the money, I can buy daily needs. So I have a better family life. (…) Moreover, I have the feeling of being equal when I talk with my mother-in-law, she does not compare me again with her other daughter-in-law. We can also spend our time going on picnics.”* (PCN126, Woman 33 years)

The study participants sometimes talk about an improvement in their life in general. They use words as a “better life” or get “my life back”.

### How was this impact reached?

Stigma was an important problem for many people affected by leprosy in Cirebon District and the counselling intervention had a positive impact on their lives. How was this impact achieved and how did the different aspects of the intervention (raising knowledge, awareness of rights, involvement of lay and peer counsellors, combination of individual, family and group counselling) contribute to the changes? In the introduction several questions were raised, which we address here.

#### Does an emphasis on knowledge work?

Clients made clear in the PCN how much they benefited from the medical information they received during the counselling. During one of the first sessions, clients’ deeply held beliefs were challenged. The PCN and CRN show there was time to share and discuss information and clients felt free and at ease to ask questions and to confirm that they had understood the information correctly. Key aspects were learning about the causes of leprosy, infectiousness and the ability to be cured. Instead of perceiving themselves as infectious and uncured, they understood and believed that they were cured and no longer infectious. This was perceived as a “miracle”, which created a space and momentum for change. Many said that this knowledge was the main reason why stigma reduced.

#### Is the awareness of rights as powerful as anticipated?

The space and momentum for change that was created through the new knowledge was strengthened with an awareness of rights, emphasised in each of the three types of counselling session. The realisation that persons affected by leprosy enjoy the same human rights as everyone else was powerful. Not only were their rights as a patient addressed, such as having access to information and treatment, but also the right to be part of family and community life, including having an education and employment. From the CRN we can deduce that clients who are aware of their rights are more confident and less afraid to take initiatives than those without this awareness.

*“Counselling has made me aware of my rights to go out*, *to make friends*, *to enjoy daily activity without feeling afraid and worried*.*”* (IDI3, Man 28 years).

#### What challenges emerged?

Although the lay and peer counsellors attended 56 hours of training, practised ten hours of counselling and had 12 hours of booster training, they still had limited experience and needed intensive supervision initially. At times it was not easy for them to manage clients’ conditions and characteristics (e.g. introvert/silent clients, clients who were bored, clients with a strong personality, clients who requested financial support), particular family behaviour (e.g. a family that rejects the client or is worried about reactions from others), creating commitment for the next session and seeing the RBCM not as a mere blueprint. In their FGD, health professionals identified two of these as potential obstacles to effective counselling. Another challenge was the wish of persons affected by leprosy or their family to conceal the illness. Some clients preferred their own house as the location for counselling, but others did not.

*“I do not need to talk, I prefer staying away and keeping quiet. Even my wife, she does not need to know about my disease and my problem.”* (IDI 8, Man 52 years).

*“Our privacy is disturbed, and too many visits also make us more afraid of our neighbours.”* (FGD2 Young clients)

*“I do not mind if you come and talk to my husband, but not too often and not too many people.”* (FGD6 Family members)

Counsellors’ inexperience and some clients’ wish to conceal their condition may well have influenced the effectiveness of the counselling provided in the SARI project.

#### Are lay and peer counsellors appreciated as counsellors?

Clients were also asked which type of counsellors they preferred. Eighty-two of 163 participants (50.3%) preferred peer counselling, 63 (38.7%) preferred leprosy workers, and 20 (12.3%) preferred the lay counsellors from a DPO. Reasons for choosing a peer counsellor were sharing similar life experiences, not feeling alone with the disease, feeling more free to talk, feeling motivated and having a role model. The benefits of peer counsellors were also described in the CRN. The counsellors noticed that they became a role model for their clients. Just by doing their job (seeking out clients, meeting new people, sharing information, sharing experiences without feeling worried or ashamed) they displayed confidence and this stimulated and motivated the clients to see things more positively than before.

*“My client was surprised that a person affected by leprosy came to her house. She stayed at home all the time, her family did not allow her to go out even though she has been cured for a long time. The arrival of the peer [counsellor] directly motivated her. Spontaneously she went out from home to the market, that she had been missing year after year.”* (FGD 7 Lay and peer counsellors)

Some clients preferred a leprosy worker was because of their expertise regarding leprosy and medication. Some wanted to make sure they were receiving the proper medical treatment, while others were quite explicit about not wanting to be counselled by a leprosy worker because of the medical focus:

*“A health worker only works for medical treatment asking to routinely take medicine without understanding our feeling, our thought and our other needs beside medication.”* (FGD 3 Elderly clients)

Reasons for preferring counselling from a lay person involved in a DPO were feeling part of the wider community, feeling inspired by people with disabilities and the information beyond the disease (leprosy, medication and treatment) on topics such as jobs, microfinance and fun/social activities.

*“We can share job opportunities, also we can get information about micro finance to run a business. By having a business and job, we do not care about stigma, we care about our work.”* (FGD5 Clients who also joined the SED intervention)

#### Is three types of counselling (family, individual and group) not too ambitious?

Each type of counselling brought something different to the process. What worked best depended largely on the individual situation and the client’s needs. Family counselling was most appreciated. Seventy-eight of the 163 RBCM clients (47.9%) wrote in their PRN that they had benefited most from the family counselling, which is very high given that only 98 clients received it. A health professional thought that this was because the family is in contact with the “patient every day, every minute, every second”. In the FGD family members and clients mentioned as reasons that counselling increased knowledge of leprosy and encouraged family members to accept, support and perhaps most importantly involve clients in family and community life:

*“Because of the family counselling my wife understands me and gives me support, she talks to me more frequently, asks me to do some household tasks.”* (FGD4 Clients with impairments)

*“After we had family counselling, we stopped avoiding our family members who have leprosy, we are not afraid to be close and talk to them, we are aware that they are part of our family and rather than excluding and avoiding them, we ask them to help us.”* (FGD6 Family members)

In total, 52 of 163 (31.9%) clients wrote that they received most benefits from individual counselling because of a feeling of comfort, confidentiality, the secure environment, space to talk and be listened to, receiving spirit and motivation to do things, communicating and advocating for rights:

*“I choose individual counselling, I need confidentiality, talking more freely and more openly.”* (IDI15, Woman 33 years)

*“By individual counselling, I feel there is a person who pays attention and cares for me, feeling (…) [of being] listened to.”* (IDI24, Woman 43 years)

Last, for 35 of 163 participants (21.5%) group counselling created the greatest benefit, although only 89 clients received group counselling. Reasons mentioned were sharing and learning from each other, being motivated by others and by the group support:

*“Really, group counselling helped us to reduce our negative feeling by talking and sharing.”* (FGD1 Clients women)

The next quote comes from the same girl who was quoted at the start this paper and illustrates nicely the added value an integration of three types of counselling brings:

*When the counsellors came to visit me I was really happy. … The conversations with the counsellors reduced my lonely feelings. Through family counselling, my parents and I are not worried anymore. (…) Conversation in the group really opened my mind. The knowledge on leprosy—that is my disease—makes me feel more released. … I have confidence to explain if people around ask me about the spot on my skin. The disease may attack my body but it does not make me lose my rights. I am not ashamed if people look at me and I am not sad if people avoid me. I ignore them and I am comfortable and able to study till high education, so later people will not look at me negatively. It is better people know rather than hide it. After I explain my disease confidently, gossiping disappears. It is my action for my future.”* (PCN89, Girl 15 years)

The findings show that integrating three types of counselling contributed to its effectiveness. The counselling skills and attitudes that the lay and peer counsellors needed for each type overlapped somewhat, but there were also important differences (e.g. facilitating a peer group versus individual counselling). This integration is ambitious, but not too ambitious, and seems essential to achieve the impact desired.

## Discussion

This paper showed that the counselling intervention was highly effective in reducing internalized stigma, creating hope and stimulating action in people affected by leprosy in Cirebon District, Indonesia. In the literature, counselling is often identified as a promising stigma-reduction approach [14,15] and this study confirms this.

Can only five counselling sessions be effective? In this study, heightened knowledge became the first trigger for change. Perceiving oneself as cured and not infectious can be described as a “miracle”. Here an interesting link can be made with the Brief Solution Based Therapy (BSBT) (a CBT variation), developed by Berg, Shazer and colleagues in the late 1970s [46]. In this approach clients are asked to imagine their life if a miracle had happened overnight. These imagined changes are taken as a starting point to find out how much of a miracle has already happened in their daily life. In the BSBT the miracle is a mental construction—a means to envisage a preferred situation. In the case of clients affected by leprosy-related stigma, knowledge about their cure functioned as genuine miracle, not an imaginary dreamlike one. So knowledge has played an essential role and an important trigger in the progress of the counselling.

The second trigger was awareness of rights. These triggers, combined with increased understanding and support from family members and real-life role models who shared their experiences, proved to be a powerful approach. Frequently, five sessions were enough to set a larger process of change in motion. The key elements of the counselling sessions thus were its i) knowledge-based approach; ii) rights-based approach; iii) CBT-based approach; iv) the integration of three types of counselling; v) lay and peer counsellors providing the counselling; vi) energetic and optimistic counselling style that combines depth and pace. Individual elements have been studied before. Floyd-Richard and Gurung [47] have, for instance, shown that group counselling can be effective for people affected by leprosy. Peters et al. [3] recommended that leprosy services should make better use of support in particular from a spouse or parents. But to our knowledge these and other elements have not previously been integrated in one brief and energetic counselling approach.

Some specific findings need further explanation. For instance, we found a change in the control area. This can be partly explained by time (we assume that some people affected by leprosy learn to live with their disease and the stigma), the data collection (which is also a form of intervention), and the attention to leprosy and stigma at district level due to the SARI project. The difference at baseline between the counselling group and the controls can be explained by bias. Only people with more severe problems would seek counselling. People with no problems were either not even offered counselling, or are likely to have refused. The control group included everyone. Some clients we offered more than just counselling and this might lead to an overestimation but also an underestimation of the results. The findings also suggest that the counselling intervention had a different impact on the pilot clients who became peer counsellors. There was a greater reduction of stigma, which is not surprising given that they joined numerous relevant activities (e.g. counselling training, regular meetings) and because of their new role and status as peer counsellors. In contrast, their perceptions regarding participation restrictions and quality of life did not improve at the same pace. It could be that their ‘peer group’ changed and that their wishes and needs in terms of quality of life evolved. We are measuring perceptions and these can alter when the context changes. This should be kept in mind when applying scales that assess perceptions. We also found that counselling had different impacts on stigma and participation restrictions on men and women. Studies have shown that women and men experience stigma differently [2,48–50], so it follows that an intervention will also have differing effects. Our hypothesis is that women and men undergo a different kind of change: through counselling men are inspired to take action and do things (e.g. talk to neighbours, find a job), whereas women go through an internal change process (e.g. change their perceptions). This would explain the difference on the SSS, but less so on the PSS. We are not aware of other studies that have found a different impact of counselling on stigma among men and women. Future research could focus on understanding the reasons in more depth and perhaps also tailor interventions accordingly.

The results are positive, but there remain important challenges. The intervention is not appropriate for everyone affected by leprosy—some people with presumably high levels of stigma refused the intervention, and the wish to conceal the disease was another barrier. So multiple interventions to address all needs are needed, a case which has also been argued for by others [14,20,51].

We recommend future research in several directions. This study showed that some people affected by leprosy are dealing well with stigma without the need of interventions. Studying why this is could lead to valuable insights for reducing stigma. Negative effects due to a lack of knowledge (e.g. self-isolation, fear, worry) could have been prevented had health professionals provided medical information at the time of diagnosis or during the treatment phase. While ignorance is a major challenge to heath care in general and in the field of leprosy specifically [8,52–54], it should remain a central topic in leprosy research. The qualitative data shows that there is an impact on stigma in the family context. It would have been useful to test this effect with quantitative measures, but more research in this field is needed. The selection, training and supervision of the lay and peer counsellors was crucial. They required specialist knowledge and it was still difficult for some counsellors to become effective [37], pointing to the need for more research on how to improve the selection and training of lay and peer counsellors. A counselling intervention needs to be sustainable, and since this intervention proved to be less sustainable than we hoped it will be vital to strengthen effective links between health professionals and lay and peer counsellors. Studies could help to establish how this can be achieved.

The key elements of the counselling seem to be appropriate to address leprosy-related stigma not only in Indonesia, but also elsewhere. During the design of the counselling intervention costs were considered, as we realised that a costly intervention would not be sustainable in a context were resources and time are scarce. In general, the execution of the intervention is inexpensive because of the involvement of lay and peer counsellors who live near the clients. Some costs are involved and some time investments need to be made for example for the training and supervision of the lay and peer counsellors. The intervention is relatively easy to replicate and scale up, so that lay and peer counsellors could play a key role in nationwide programmes to combat leprosy-related stigma. Lay counsellors need to be trained not only in understanding human rights and appropriate counselling skills but also to observe a code of conduct in dealing with marginalized and vulnerable clients. To enable them to assist their clients effectively, the lay counsellors should also receive a clear (written) mandate from the health authority to facilitate clients’ access relevant to public facilities including the social protection schemes currently promoted by the local and central government. The findings of this study are also relevant for other stigmatized conditions. Several Neglected Tropical Diseases (NTD) are associated with stigma (e.g. Buruli ulcer, lymphatic filariasis, onchoceriasis, leishmaniasis and Chagas disease) and so have negative effects on an individual’s quality of life [24,55–57]. The value of counselling will depend on identifying similar triggers for change. For example, the knowledge element regarding HIV/AIDS could be that the condition is not as infectious as people often believe, or in the field of NTD that some diseases can be treated or managed with medication.

### Conclusion

Rafferty [12] stated that “if patients are cured, the stigmatization can remain an insurmountable obstacle to the resumption of a normal life”. This study shows that the obstacle is not insurmountable. The findings demonstrate that the counselling intervention is effective in decreasing stigma, promoting the rights of people with leprosy and in facilitating their participation in family and community life. We recommend its application on a larger scale. More research is needed to create a more sustainable implementation of the counselling, preferably structurally embedded in the health or social services.

## Supporting Information

S1 Checklist STROBE Checklist. (PDF)
